# Effect of positive end-expiratory pressure on pulmonary compliance and pulmonary complications in patients undergoing robot-assisted laparoscopic radical prostatectomy: a randomized control trial

**DOI:** 10.1186/s12871-022-01869-1

**Published:** 2022-11-12

**Authors:** Menglan Cheng, Lifeng Ni, Ling’er Huang, Yanfeng Zhou, Kuirong Wang

**Affiliations:** Department of Anesthesiology, The First Affiliated Hospital, Zhejiang University School of Medicine, Hangzhou, Zhejiang China

**Keywords:** Robot-assisted laparoscopic radical prostatectomy, Pulmonary compliance, Positive end-expiratory pressure

## Abstract

**Background:**

To observe the effects of different positive end-expiratory pressure (PEEP) ventilation strategies on pulmonary compliance and complications in patients undergoing robotic-assisted laparoscopic prostate surgery.

**Methods:**

A total of 120 patients with the American Society of Anesthesiologists Physical Status Class I or II who underwent elective robotic-assisted laparoscopic prostatectomy were enrolled. We randomized the patients divided into divided into three groups of 40 patients each: PEEP0, PEEP5, or PEEP10. Master Anesthetist used volume control ventilation intraoperatively with an intraoperative deep muscle relaxation strategy. Respiratory mechanics indexes were recorded at six time-points: 10 mimuts after anaesthesia induction, immediately after pneumoperitoneum establishment, 30 min, 60 min, 90 min, and at the end of pneumoperitoneum. Arterial blood gas analysis and oxygenation index calculation were performed 10 mimuts after anaesthesia induction, 60 mimuts after pneumoperitoneum, and after tracheal extubation. Postoperative pulmonary complications were also recorded.

**Results:**

After pneumoperitoneum, peak inspiratory pressure (Ppeak), plateau pressure (Pplat), mean pressure (Pmean), driving pressure (ΔP), and airway resistance (Raw) increased significantly, and pulmonary compliance (Crs) decreased, persisting during pneumoperitoneum in all groups. Between immediately after pneumoperitoneum establishment, 30 min, 60 min, and 90 min, pulmonary compliance in the 10cmH_2_OPEEP group was higher than in the 5cmH_2_OPEEP (*P* < 0.05) and 0cmH_2_OPEEP groups(*P* < 0.05). The driving pressure (ΔP) immediately after pneumoperitoneum establishment, at 30 min, 60 min, and 90 min in the 10cmH_2_OPEEP group was lower than in the 5cmH_2_OPEEP (*P* < 0.05) and 0cmH_2_OPEEP groups (*P* < 0.05). Sixty min after pneumoperitoneum and tracheal extubation, the PaCO_2_ did not differ significantly among the three groups (*P* > 0.05). The oxygenation index (PaO_2_/FiO_2_) was higher in the PEEP5 group than in the PEEP0 and PEEP10 groups 60 min after pneumoperitoneum and after tracheal extubation, with a statistically significant difference (*P* < 0.05). In postoperative pulmonary complications, the incidence of atelectasis was higher in the PEEP0 group than in the PEEP5 and PEEP10 groups, with a statistically significant difference (*p* < 0.05).

**Conclusion:**

The use of PEEP at 5cmH_2_O during RARP increases lung compliance, improves intraoperative oxygenation index and reduces postoperative atelectasis.

**Trial registration:**

This study was registered in the China Clinical Trials Registry on May 30, 2020 (Registration No. ChiCTR2000033380).

**Supplementary Information:**

The online version contains supplementary material available at 10.1186/s12871-022-01869-1.

## Background

The International Agency for Research in Oncology (IARC) estimated 1,414,259 new cases of prostate cancer and approximately 375,304 prostate-cancer-related deaths worldwide in 2020 [1]. With the continuous advancement of minimally invasive surgery and the rapid development of artificial intelligence-assisted systems, an increasing number of studies have shown that robotic-assisted laparoscopic radical prostatectomy (RARP) is superior to traditional open radical prostatectomy or pure laparoscopic radical prostatectomy in several aspects, such as providing a more specific field, a more delicate operation execution, less trauma, less blood loss, and complete revolutionary treatment [2]. While robot-assisted surgery has benefited prostate cancer patients, the anaesthetic management of patients undergoing RARP surgery, especially managing the physiological changes due to pneumoperitoneum and a vertical head-down position, has become one of the main recent topics in anesthesiology [3, 4]. The establishment of pneumoperitoneum and head-down position can cause serious interference with pulmonary function: first, it affects diaphragm elevation, causing decreased thoracopulmonary compliance, reduced functional residual air volume, and pulmonary atelectasis. This increases the possibility of hypoxemia. Ventilation pressure also rises significantly, which may damage the lungs and increase the occurrence of postoperative pulmonary dysfunction. Pulmonary dysfunction occurs in approximately 5% of patients undergoing surgical procedures under general anaesthesia with tracheal intubation, leading to prolonged postoperative recovery and increased hospital costs [5]. There are many causes of postoperative pulmonary complications, including barotrauma during general anaesthesia [4, 5]. Therefore, perioperative pulmonary protection anaesthetic management is essential to rapid patient recovery. Pulmonary protective ventilation, which combines a low tidal volume (6–8 mL/kg) and positive end-expiratory pressure (PEEP) ventilation, was initially used in patients with respiratory distress syndrome and is now considered beneficial in "healthy lungs" patients under general anaesthesia with tracheal intubation [5, 6]. For laparoscopic procedures requiring CO2 pneumoperitoneum, a "permissive hypercapnia," where small tidal volume ventilation is applied and the arterial blood CO2 partial pressure is permitted to reach ≥ 60–70 mmHg for a short period, was proposed to avoid lung damage from high ventilation pressure [6]. In a study of 40 patients who underwent elective abdominal surgery with individual PEEP value monitoring by thoracic image scanning, a PEEP of 6–16 cmH_2_O with a median of 12 cmH_2_O was required to improve pulmonary compliance with pulmonary atelectasis [7]. It has also been suggested that high PEEP levels (10 cmH_2_O) significantly improve lung compliance and reduce the incidence of atelectasis during mechanical ventilation compared to low PEEP levels and no PEEP ventilation [8, 9].

Most anaesthetized patients treated with RARP surgery have a healthy level of pulmonary function with good lung compliance. There is a lack of systematic studies on whether intraoperative PEEP is required to improve oxygenation and reduce postoperative pulmonary complications in this group. It is essential to guide clinical anesthesiologists in managing the respiratory function of patients undergoing RARP surgery with safer and more effective mechanical ventilation parameters by identifying the appropriate PEEP values. In this study, we investigated the feasibility of a PEEP ventilation strategy for patients undergoing RARP surgery and its effects on ventilation, oxygenation, and Postoperative oxygenation function.

### Patients and methods

This prospective randomized, controlled trial was reviewed and approved by the IIT Ethics Review Panel of the Clinical Research Ethics Committee of the First Hospital of Zhejiang University School of Medicine on 06/05/2020 (Session No. 48). The study was registered in the China Clinical Trials Registry (Registration No. ChiCTR2000033380).

Our research follows the ethical standards of the WHO Declaration of Helsinki (1964) and its successive amendments. Studies that are adequately controlled, blinded, randomized, and of sufficient statistical power to confidentially and accurately interpret the effect reported.

### Patients

**Fig. 1:**
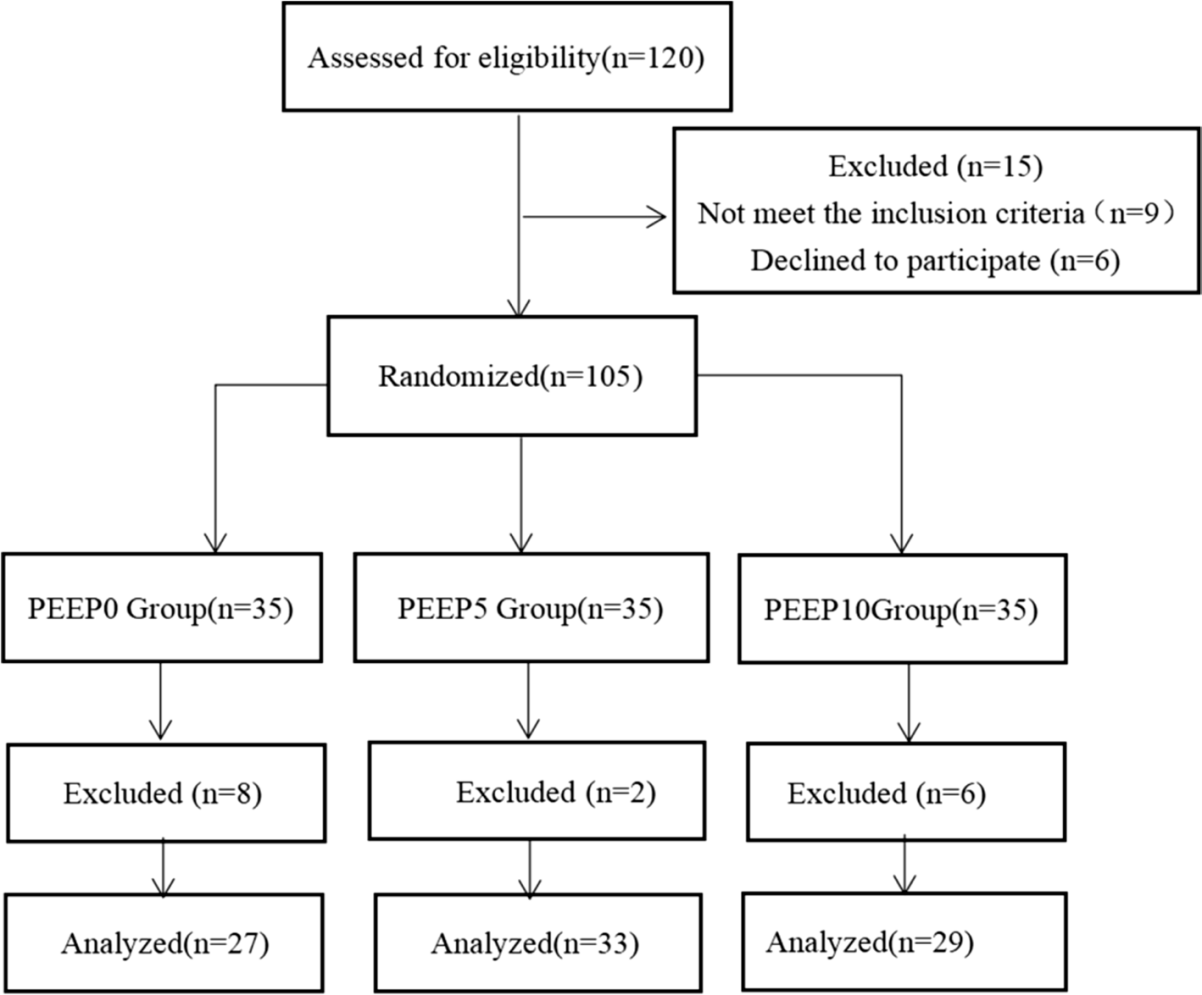
Experimental flow chart

We recruited a total of 120 patients who underwent robotic-assisted laparoscopic radical prostatectomy at the First Hospital of Zhejiang University School of Medicine from July 2020 to June 2021. Written informed consent was obtained from all participants. The inclusion criteria were Patients undergoing RARP, ASA classification I-III. Exclusion criteria were age > 80 years, history of severe cardiopulmonary, hepatic, and renal disease, history of neuromuscular disease, excessive obesity or malnutrition (body mass index, BMI ≥ 30 or ≤ 20), and history of drug allergy. Hemodynamic instability due to positive end-expiratory pressure during the study period and difficulty in maintaining mean arterial pressure above 65 mmHg with intravenous norepinephrine (0.03ug/kg/h) will be terminated. Using SPSS 23 (IBM, Armonk, NY, USA), patients were randomly allocated to three groups (40 patients per group): PEEP0, PEEP5, and PEEP10 groups. Randomization was performed by a researcher not involved in the anesthesia or statistical analysis. The attending anesthetist was given an envelope containing the allocation results. The patient, the surgeon, and the resident anesthetist responsible for the records were blinded to the PEEP level. Nine patients did not meet the inclusion criteria, and six declined to participate. Eight patients were excluded due to issues with recruitment, loss of data records, and loss of follow-up, four patients were excluded due to a change of surgical approach, two patients were terminated due to failure to maintain circulation, and 2 cases were excluded due to failure of pneumoperitoneum time. A flowchart of the study is shown in Fig. 1.

### Anesthesia method

Patients were routinely monitored after admission to the operating room, arterial pressure was measured continuously by proper radial artery puncture placement, and we performed blood gas analysis. Anaesthesia was induced intravenously with etomidate 0.3 mg/kg, fentanyl 4 μg/kg, and rocuronium 0.6 mg/kg, followed by tracheal intubation. All patients were mechanically ventilated using a DrägerFabius GS anaesthesia workstation ( Dräger Medical Center, Lübeck, Germany) with ventilation set to volume-controlled breathing (60% oxygen concentration, 1:1 air mixture), tidal volume set to an initial value of 7 ml/kg based on ideal body weight, frequency 12 times/min, and PEEP set to 0, 5, and 10 cmH_2_O, respectively, according to randomized groups. The Anaesthetist adjusted the respiratory frequency before pneumoperitoneum (Pnp) to keep the partial pressure of end-expiratory carbon dioxide (E_T_CO_2_) at 30–35 mmHg. IfE_T_CO_2_ ≤ 60 mmHg, Anaesthetist did not adjust the respiratory parameters, but if E_T_CO_2_ > 60 mmHg, the increase in respiratory frequency was first adjusted. If E_T_CO_2_ continued to rise, the tidal volume was increased appropriately. According to the pretest, Ppeak was generally less than 30cmH_2_O water column. In addition, we set 40cmH_2_O as the upper limit to prevent unacceptable levels of high driving or plateau pressure. Anaesthetists set no recruitment manoeuvres for any ventilation modes.

At the beginning of surgery, the Anaesthetist adjusted the position to a 30-degree head-down position according to the surgical needs, and the pneumoperitoneum pressure was adjusted to maintain 13 cmH20.

Anaesthesia maintenance: propofol 4–10 mg/kg/h and remifentanil 8–18 μg/kg/h pumped to keep the BIS value at 40–60. We administered intraoperative rocuronium bromide at 0.6 mg/kg/h to maintain a deep neuromuscular block (NMB). Deep NMB is defined as no responses to train-of-four (TOF) stimulation and 1–2 replies to post-tetanic count (PTC) during neuromuscular monitoring. After pneumoperitoneum, the surgery did not require profound neuromuscular blockade, and we no longer recorded pulmonary compliance indicators as we did in the state of profound neuromuscular blockade. Rocuronium was discontinued at the end of the pneumoperitoneum.

At the end of the surgery, neostigmine (0.05 mg/kg) and atropine (0.1 mg/kg) was intravenously administered under the guidance of the NMB monitor. The patient was extubated in the anaesthesia recovery room (PACU), and Travelling Nurse performed a blood gas analysis before Recovery Room Nurse sent the patient to the general ward. The criteria for discharge from the PACU was an Aldrete score of > 9, as assessed by the anesthesiologist in charge of the PACU before leaving the PACU and returning to the ward. Patients performed a low-dose chest computed tomography (CT) scan the day after the operation. **2.3.** *Monitoring items and time points.*

We monitored patients with ECG, pulse oximetry, temperature, BIS, invasive arterial pressure, E_T_CO_2_, and ventilation pressure–volume loop using a CARESCAPE Monitor B650 (GE Medical, Helsinki, Finland) monitor. Blood gas analysis was performed using an ABL-90FLEX analyzer (ApS, Brønshøj, Denmark), and neuromuscular blockade was monitored by accelerated EMG of the thumb adductors using TOF Watch SX (Olga, Dublin, Ireland).

The primary outcome indicators were comparing the effects of applying 0, 5, and 10 cmH_2_OPEEP on pulmonary compliance (Crs) and driving pressure (ΔP) during pneumoperitoneum in patients undergoing RARP. The secondary outcomes of the study were oxygenation index and pulmonary complications.

Team personnel recorded data at the following six time-points for PEEP0, PEEP5, and PEEP10 groups: after induction, pneumoperitoneum establishment, 30 min after pneumoperitoneum, 60 min after pneumoperitoneum, 90 min after pneumoperitoneum, and at the end of pneumoperitoneum: tidal volume, respiratory rate, end-expiratory carbon dioxide partial pressure, peak airway pressure, plateau pressure, lung compliance, airway resistance, finger oxygen saturation, blood pressure, heart rate, and duration of surgery. Anaesthetist performed blood gas analysis after induction of anaesthesia, 1 h after pneumoperitoneum, and after tracheal extubation. Tracheal extubation time, PACU time, and agitation during the awakening period were recorded in the PACU. Tracheal extubation time was the time from the end of surgery to the tracheal tube removal. PACU time was the time from admission to departure from the PACU. PACU anaesthesia nurses recorded the patient's Riker score; if the Riker score was ≥ 5, we called it awakening agitation.

Follow-up visits were performed on postoperative days 1 and 3 and 1 month postoperatively to record postoperative pain scores, finger pulse oxygen saturation, and postoperative complications.

### Statistical analyses

The Kolmogorov–Smirnov test was used to test the normality of the distribution of all variables. Values for peak inspiratory pressure (Peak), mean pressure (Pmean), lung compliance (Crs), airway resistance (Raw), partial pressure of carbon dioxide in the arteries (PaCO2) and the ratio of partial pressure of O2 in the arterial blood to the fraction of oxygen absorbed (PaO2/FiO2) at different time points are expressed as mean and standard deviation. Patient characteristics, time to pneumoperitoneum, time to surgery and time to extubation are expressed as means and standard deviations. One-way ANOVA was used to analyse differences between groups for normally distributed measures, and LSD tests were used for post hoc two-way comparisons. Differences between multiple time points for non-normally distributed actions were analysed using Kruskal–Wallis and one-way analysis of variance (ANOVA) post hoc tests, and Mann–Whitney U tests were used to analyse differences between the two-time points and groups. χ2 tests were used to compare the number of patients with agitation on awakening and the number of patients with postoperative pulmonary complications in all groups.

A pre-test was performed to determine the sample size. The mean Crs at PEEP for pneumoperitoneum (Pnp) 0, 5 and 10 cmH_2_O were 27 mL/cmH_2_O, 32 mL/cmH_2_O and 34 mL/cmH_2_O with standard deviations of 7, 9 and 10 respectively. considering a P value = 0.05 and a degree of certainty of 0.90, a minimum of 28 patients per group was required to differentiate the Crs in each group.

## Results

Patient characteristics such as age, BMI, time to pneumoperitoneum and time to surgery (Table 1) did not differ significantly between the groups.

**Table 1:** Basic patient characteristics

	**PEEP0**	**PEEP5**	**PEEP10**	***P*-value**
N	27	33	29	
Age (years)	66 ± 5	69 ± 7	67 ± 8	0.089
BMI (kg/m2)	24.1 ± 2.0	23.2 ± 2.2	23.6 ± 2.3	0.452
Surgerytime(min)	157.2 ± 12.1	159.5 ± 12.5	155.6 ± 13.2	0.446
Pneumoperitoneum time (min)	127.3 ± 10.2	129 ± 11.5	125.4 ± 12.6	0.514
ASA Classification (Class II/III)	15/12	14/13	13/14	0.891

*P* value is the analysis of variance comparison between the three groups

Comparison of haemodynamic values between the three groups of patients. For mean arterial pressure(MAP), there was no statistically significant difference between the three groups at each time point; compared between the three groups, MAP values in the T2.T3.T4.T5 PEEP10 group were lower than those in the PEEP0 and PEEP5 groups, with statistically significant differences (*p* < 0.05) (Table 2). There was no significant difference in heart rate (HR)between the three groups of patients, between and within group comparisons(*p* > 0.05) (Table 2).

**Table 2:** Hemodynamic changes in three groups

		**T1**	**T2**	**T3**	**T4**	**T5**	**T6**
MAP (mmHg)	PEEP0	85.8 ± 7.7	88.4 ± 7.2	87.3 ± 5.4	88.0 ± 5.6	88.2 ± 6.1	92.1 ± 6.2
	PEEP5	84.8 ± 5.8	87.6 ± 6.9	86.4 ± 4.2	85.9 ± 6.2	86.1 ± 5.8	91.5 ± 5.8
	PEEP10	85.6 ± 6.7	81.8 ± 7.1^ab^	81.4 ± 5.2^ab^	82.4 ± 5.9^ab^	81.5 ± 6.1^ab^	91.7 ± 5.6
HR (Times/minute)	PEEP0	60.2 ± 6.1	59.9 ± 6.7	59.0 ± 5.1	60.4 ± 5.7	61.2 ± 6.1	71.6 ± 8.3
	PEEP5	59.8 ± 5.8	61.6 ± 7.1	58.8 ± 6.2	61.1 ± 5.2	62.2 ± 5.6	71.2 ± 7.5
	PEEP10	59.6 ± 5.6	60.5 ± 6.0	60.7 ± 5.0	59.4 ± 6.4	60.2 ± 4.5	72.1 ± 7.2

*T1* post induction, *T2* immediate post pneumoperitoneum, *T3* 0.5 h post pneumoperitoneum, *T4* 1 h post pneumoperitoneum, *T5* 1.5 h post pneumoperitoneum, *T6* end of pneumoperitoneum

^a^Compared with the PEEP0 group, *P* < 0.05

^b^Compared with the PEEP5 group, *P* < 0.05

**Fig. 2:**
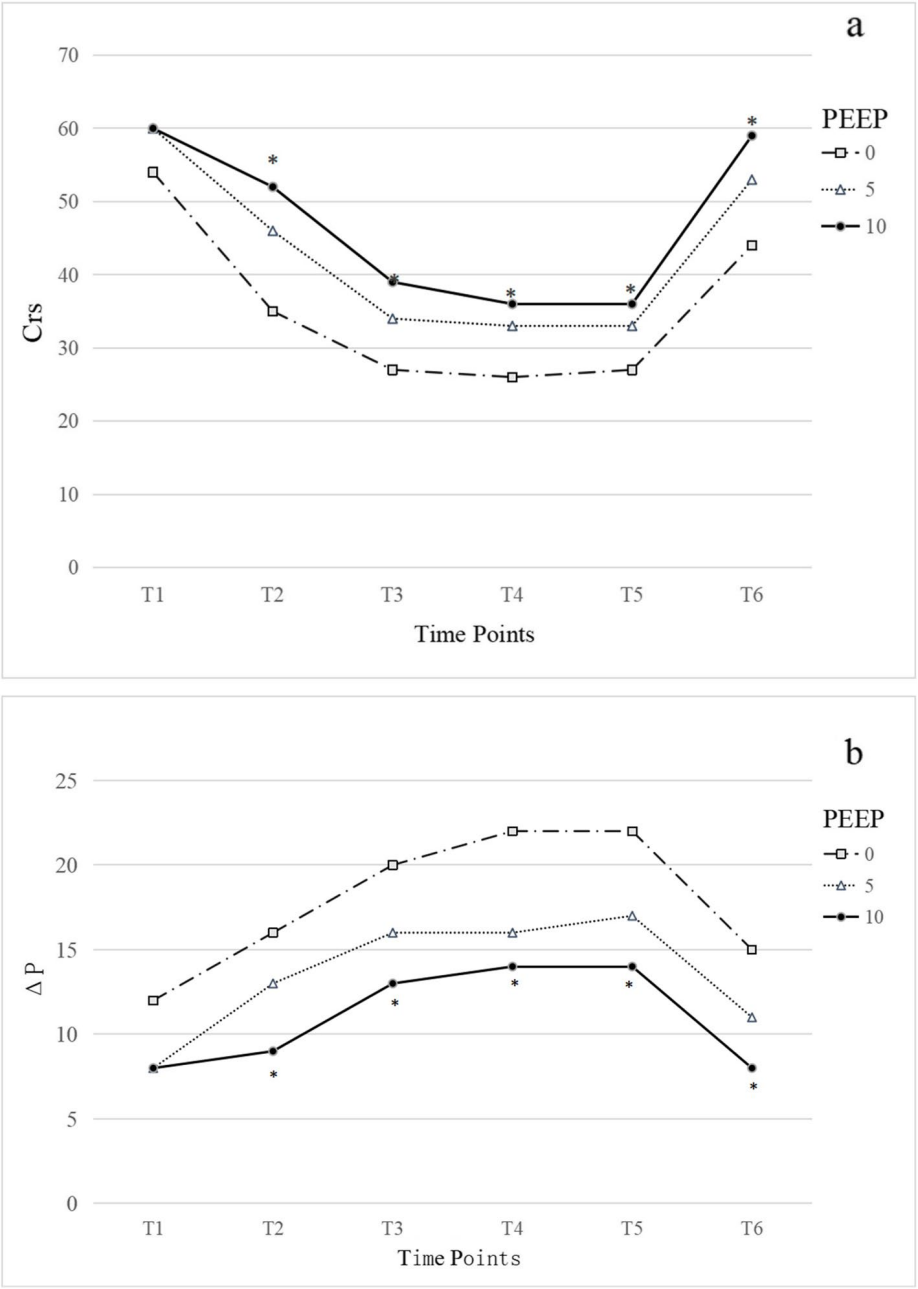
Respiratory mechanics at different time points during surgery

After pneumoperitoneum establishment, Peak, Pmean, Raw, Plat and ΔP (driving pressure) increased, while Crs decreased (Additional file 1: Supplementary Tables 1, 2 and 3). These changes persisted in all groups of pneumoperitoneum. These indices improved at the end of the pneumoperitoneum but did not return to post-induction levels. Shortly after pneumoperitoneum establishment, between 30, 60 and 90 min, lung compliance was higher in the PEEP10 group than in the PEEP5 group (53.7/39.2/37.2/35.8 vs 46/33.6/33.7/32.5; P < 0.05) and thePEEP0 group (53. 7/39.2/ 37.2/35.8 vs 38.4/28.2/26.7/27.4; *P* < 0.05) (Fig. 2). After establishment of the pneumoperitoneum, the driving pressure (ΔP) was lower in the PEEP10 group than in the PEEP5 group (9. 7/13.2/13.8/14.3 vs. 12.3/16.0/16.2/17.3; P < 0.05) and the PEEP0 group (9.7/13.2/ 13.8/14.3 vs. 17.0/21/22.3/22.0; *P* < 0.05) (Fig. 2). There were no significant differences (*P* > 0.05) in peak, mean and raw values for the 0 and 5 cmH_2_O PEEP groups immediately after pneumoperitoneum establishment, 30 min, 60 min, and 90 min (Additional file 1: Supplementary Tables 1, 2 and 3). The haemodynamics of the three groups at each time point were statistically lower in the PEEP10 group than in the PEEP0 and PEEP5 groups (*P* < 0.05) (Table 3). There was no significant difference in PaCO_2_ between the three groups at 60 min after pneumoperitoneum and tracheal extubation (*P* > 0.05). At 60 min after pneumoperitoneum and after tracheal extubation, the oxygenation index (PaO/FiO_2_) was higher in the PEEP5 group than in the PEEP0 and PEEP10 groups, with a statistically significant difference (*P* < 0.05) (Table 3). Extubation and PACU time was significantly longer in the 5cmH_2_O PEEP group (*P* < 0.05) (Table 4), but there was no significant difference in agitation during the awakening period between the groups (*P* > 0.05, Table 4). In terms of postoperative pulmonary complications, the incidence of pulmonary atelectasis was higher in the PEEP0 group than in the PEEP5 and PEEP10 groups, with a statistically significant difference (*P* < 0.05) (Table 5).

**Table 3:** Arterial blood gas variables at different time points

PaCO_2_(mmHg)				PaO_2_/FiO_2_(mmHg)		
	PEEP0	PEEP5	PEEP10	PEEP0	PEEP5	PEEP10
T1	42.3 ± 3.7	42.1 ± 3.9	42.9 ± 1.7	465 ± 11.2	490 ± 15.3	450 ± 13.7
T4	51.1 ± 2.5	52.2 ± 1.9	55.2 ± 1.6	425 ± 12.7	432 ± 15.3^ab^	423 ± 11.5
T7	43.2 ± 3.1	46.7 ± 2.6	45.7 ± 2.5	302 ± 13.1	363.5 ± 12.0^ab^	292 ± 16.2

*T1* induction of anesthesia, *T4* one hour after pneumoperitoneum, *T7*, after tracheal extubation

^a^Compared with the PEEP0 group, *P* < 0.05

^b^Compared with the PEEP10 group, *P* < 0.05

**Table 4:** Three groups in the post-operative recovery room

	PEEP0	PEEP5	PEEP10	**p**
Timeof extubation (min)	42.3 ± 3.9	53.5 ± 4.2^a^	41.3 ± 3.6	0.19
Time in PACU (min)	80.2 ± 8.9	92.6 ± 7.5^a^	83.7 ± 6.8	0.22
Restlessness during the awakening period (*n*)	4(14.8%)	6(18.8%)	4(13.7%)	0.865

^a^Compared with the PEEP0 group, *P* < 0.05

**Table 5:** Comparison of post-operative pulmonary complications in the three groups

		PEEP0	PEEP5	PEEP10
Pulmonary atelectasis (*n*)	5(18.5%)^ab^		1(3.0%)	1(3.4%)
Lung infection (*n*)	2(7.4%)		1(3.0%)	1(3.4%)
Pleural effusion (*n*)	1(7.4%)		1(3.0%)	0
Bronchitis (*n*)	0		0	1(3.4%)

PEEP: positive end-expiratory pressure

^a^Compared with the PEEP5 group, *P* < 0.05

^b^Compared with the PEEP5 group, *P* < 0.05

## Discussion

Laparoscopic patients under general anaesthesia are prone to pulmonary atelectasis and postoperative pulmonary complications after mechanical ventilation [11, 12]. In RARP, the incidence of atelectasis often increases due to the patient's older age and surgical position. To obtain the best surgical view, RARP requires a Trendelenburg position > 30˚, with intra-abdominal organs compressing the diaphragm and lungs. In addition, the elderly tend to have poor lung compliance and a higher incidence of postoperative pulmonary atelectasis than younger patients [10, 13]. Furthermore, our previous study showed that profound neuromuscular blockade during RARP did not increase pulmonary compliance and reduce pulmonary complications [14].

We recorded these respiratory kinetic parameters (Peak, Pmean, Crs, and Raw) at six different time points throughout the pneumoperitoneum. Therefore, our study found that the establishment of pneumoperitoneum and the change in surgical position decreased respiratory system compliance in patients, consistent with previous results [15]. This may be due to a decrease in pulmonary compliance caused by diaphragmatic elevation, restricted thoracic motion due to pneumoperitoneal pressure, and the effect of gravity in the Trendelenburg position, which increases pulmonary stasis, further disturbing pulmonary ventilation-perfusion ratio. Patients administered either 5 cmH_2_O or 10 cmH_2_O PEEP in this study showed an increase in lung compliance, presumably because a certain PEEP level counteracts the effects of manual pneumoperitoneum and the Trendelenburg position on lung compliance in RARP patients. These results are consistent with previous clinical findings [16]. Most studies on intraoperative protective mechanical ventilation have not individualized the PEEP level applied. An arbitrary selection of PEEP levels in different patient populations and surgical approaches can lead to heterogeneity among results [17]. The choice of PEEP level should be based on the patient's characteristics, the specific surgical access site, and the patient's position [18]. In a study of obese patients undergoing laparoscopic bariatric surgery, using an individualized stepwise PEEP approach with lung ultrasound improved lung compliance and oxygenation [19]. There were no significant differences in Peak, Mean, or Raw between the 0 and 5 cmH_2_O PEEP groups 10 min after anaesthesia induction, 60 min after pneumoperitoneum, or after tracheal extubation. We feel that the sample size may not have been large enough to detect statistically significant differences between the 0 cmH_2_O PEEP and 5cmH_2_O PEEP.

In patients with respiratory distress, a decrease inΔp induced by a change in ventilator settings is strongly associated with increased survival [20]. Previous findings indicate that high driving pressure in intraoperative mechanical ventilation in elective surgery is an essential factor in lung injury and is strongly associated with postoperative pulmonary complications (PPCs) [21]. Some experts proposed a lung-protective ventilation strategy for surgical patients with Δp values recommended below 14 cmH_2_O [22]. The titration method of progressively decreasing PEEP values after a pulmonary resuscitation manoeuvre improves oxygenation and reduces intraoperative ΔP. The titration method of progressively decreasing PEEP values after one pulmonary retention manoeuvre, which improves oxygenation and reduces intraoperative ΔP, was recently demonstrated in a study of 60 patients on single-lung ventilation [23]. Our results show that the driving pressure is constantly more significant than 14 cmH_2_O in the 0 cmH_2_O PEEP group. Comparing the three groups, the 10cmH_2_O group was the smallest Δp value(Δp section of Fig. 2). The 10 cmH_2_O allows for maintaining more tolerable values of driving pressure during pneumoperitoneum.

Furthermore, the results of this study demonstrate that PEEP at 10 cm H_2_O did not significantly improve key intraoperative oxygenation indices or considerably reduce the incidence of postoperative pulmonary complications but may reduce mean arterial pressure, demonstrating a limited protective effect on the lungs. Similarly, Van Hecke et al. reported that optimizing lung compliance by PEEP during laparoscopic bariatric surgery did not reduce the incidence of postoperative hypoxaemia [24]. In contrast, combining pulmonary resuscitation strategies with PEEP significantly reduced perioperative pulmonary complications compared with PEEP alone in elderly patients undergoing RARP surgery [25]. We hypothesized that appropriate PEEP would increase end-expiratory alveolar volume, reduce intrapulmonary shunts, increase lung compliance and improve oxygenation. However, inappropriately high PEEP can lead to higher airway and plateau pressures, which may produce high-pressure lung injury [26]. Excessively high PEEP levels increase thoracic pressure, which significantly affects proper ventricular outflow resistance [27], which in turn can cause an imbalance in the ventilation-perfusion ratio. Secondly, the reduction in lung area relative to the thoracic cavity produced by the Trendelenburg position and the increased elasticity of the chest wall due to the rigid frame of the robotic arm, as Future E et al. showed that appropriate PEEP could effectively reduce these effects [28,29]. Future studies should perhaps investigate and implement lung-protective queuing ventilation strategies, using measures such as electrical impedance tomography (EIT) of the chest or lung ultrasound to guide appropriate PEEP values.

There are limitations to this study. Firstly, postoperative lung function and lung CT monitoring, which would have helped to accurately assess the incidence of pulmonary complications, were not performed. Secondly, intraoperative cardiac output values were not recorded, which would have allowed us to understand the haemodynamic impact of different PEEP values. The lack of transpulmonary pressure monitoring is also a shortcoming. Thirdly, the PEEP group of 0 cmH_2_O did not reach the intended number of 28 patients. Finally, we did not individualize the PEEP settings [30]. Using individualized titrated PEEP settings for perioperative lung protection may be a future trend.

## Conclusion

In robotic-assisted laparoscopic prostatectomy under deep muscle relaxation, the application of PEEP at 5 cm H_2_O level not only increases lung compliance, reduces driving pressure, improves intraoperative oxygenation index and reduces pulmonary atelectasis but also has less impact on blood pressure compared to PEEP at 0 cm H_2_O and 10 cm H_2_O levels. An individualized lung-protective ventilation strategy is required in the future.

## Supplementary Information

**Additional file 1:** **Supplementary** **Table 1.** Intra-group situation of the 0 cmH_2_O PEEP group at different time points. **Supplementary** **Table 2** Intra-group situation of the 5 cmH_2_O PEEP group at different time poins. **Supplementary** **Table** **3** Intra-group situation of the 10cmH_2_O PEEP group at different time poins.

## Data Availability

All data generated or analyzed during this study are included in its supplementary information files.

## Abbreviations

PEEP: Positive end-expiratory pressure
Ppeak: Peak inspiratory pressure
Pplat: Plateau pressure
Pmean: Mean pressure
ΔP: Driving pressure
Raw: Airway resistance
Crs: Pulmonary compliance
PaCO_2_: Partial pressure of carbon dioxide
PaO_2_: Partial pressure of oxygen
