# Supplementary material for: Effect of positive end-expiratory pressure on pulmonary compliance and pulmonary complications in patients undergoing robot-assisted laparoscopic radical prostatectomy: a randomized control trial
Source: BMC Anesthesiol. 2022 Nov 12;22:347. doi: 10.1186/s12871-022-01869-1 (PMC9652925; doi:10.1186/s12871-022-01869-1)
Supplement: Supplementary file 1 — Additional file 1: Supplementary Table 1. Intra-group situation of the 0 cmH2O PEEP group at different time points. Supplementary Table 2 Intra-group situation of the 5 cmH2O PEEP group at different time poins. Supplementary Table 3 Intra-group situation of the 10cmH2O PEEP group at different time poins. [file 12871_2022_1869_MOESM1_ESM.docx]

**Supplementary Table 1: Intra-group situation of the 0 cmH_2_O PEEP group at different time points .**

| Time Point | *P*-value | | | | | |
| --- | --- | --- | --- | --- | --- | --- |
|  | **Crs** | **Raw** | **Ppeak** | **Pmean** | **Plat** | **ΔP** |
| T1-T2 | 0.008 | 0.234 | 0.017 | 0.109 | 0.007 | 0.008 |
| T1-T3 | 0.000 | 0.000 | 0.000 | 0.000 | 0.000 | 0.000 |
| T1-T4 | 0.000 | 0.002 | 0.000 | 0.000 | 0.000 | 0.000 |
| T1-T5 | 0.000 | 0.000 | 0.000 | 0.000 | 0.000 | 0.000 |
| T1-T6 | 1.000 | 1.000 | 0.125 | 0.123 | 0.656 | 0.784 |
| T2-T3 | 0.003 | 0.133 | 0.006 | 0.119 | 0.006 | 0.010 |
| T2-T4 | 0.004 | 1.000 | 0.001 | 0.002 | 0.002 | 0.004 |
| T2-T5 | 0.001 | 0.473 | 0.000 | 0.000 | 0.000 | 0.000 |
| T2-T6 | 0.181 | 0.719 | 1.000 | 1.000 | 1.000 | 1.000 |
| T3-T4 | 1.000 | 1.000 | 1.000 | 1.000 | 1.000 | 1.000 |
| T3-T5 | 1.000 | 1.000 | 1.000 | 0.800 | 1.000 | 1.000 |
| T3-T6 | 0.000 | 0.000 | 0.000 | 0.105 | 0.000 | 0.000 |
| T4-T5 | 1.000 | 1.000 | 1.000 | 1.000 | 1.000 | 1.000 |
| T4-T6 | 0.000 | 0.011 | 0.000 | 0.002 | 0.000 | 0.000 |
| T5-T6 | 0.000 | 0.000 | 0.000 | 0.000 | 0.000 | 0.000 |

Data are presented as the P-values for each parameter.The p-values were adjusted for multiple comparisons using the Bonferroni correction.T1 :post induction; T2:immediate post pneumoperitoneum; T3:0.5 hours post pneumoperitoneum; T4 :1 hour post pneumoperitoneum; T5:1.5 hours post pneumoperitoneum; T6:end of pneumoperitoneum; PEEP:positive end-expiratory pressure; Ppeak:peak inspiratory pressure; Plat:plateau pressure; Pmean:mean pressure; Crs:respiratory compliance; Raw:airway resistance; ΔP:driving pressure.

**Supplementary Table 2:Intra-group situation of the 5 cmH_2_O PEEP group at different time poins .**

| Time Point | *P*-value | | | | | |
| --- | --- | --- | --- | --- | --- | --- |
|  | **Crs** | **Raw** | **Ppeak** | **Pmean** | **Plat** | **ΔP** |
| T1-T2 | 1.000 | 1.000 | 0.173 | 1.000 | 0.180 | 0.156 |
| T1-T3 | 0.000 | 0.000 | 0.000 | 0.000 | 0.000 | 0.000 |
| T1-T4 | 0.000 | 0.000 | 0.000 | 0.000 | 0.000 | 0.000 |
| T1-T5 | 0.000 | 0.000 | 0.000 | 0.000 | 0.000 | 0.000 |
| T1-T6 | 1.000 | 1.000 | 1.000 | 1.000 | 1.000 | 1.000 |
| T2-T3 | 0.003 | 0.038 | 0.003 | 0.000 | 0.009 | 0.003 |
| T2-T4 | 0.000 | 0.016 | 0.000 | 0.000 | 0.001 | 0.000 |
| T2-T5 | 0.000 | 0.017 | 0.000 | 0.000 | 0.001 | 0.000 |
| T2-T6 | 1.000 | 1.000 | 1.000 | 1.000 | 0.804 | 0.905 |
| T3-T4 | 1.000 | 1.000 | 1.000 | 1.000 | 1.000 | 1.000 |
| T3-T5 | 1.000 | 1.000 | 1.000 | 1.000 | 1.000 | 1.000 |
| T3-T6 | 0.000 | 0.000 | 0.000 | 0.000 | 0.000 | 1.000 |
| T4-T5 | 1.000 | 1.000 | 1.000 | 1.000 | 1.000 | 1.000 |
| T4-T6 | 0.000 | 0.000 | 0.000 | 0.000 | 0.000 | 0.000 |
|  |  |  |  |  |  |  |
| T5-T6 | 0.000 | 0.000 | 0.000 | 0.000 | 0.000 | 0.000 |

Data are presented as the P-values for each parameter.The p-values were adjusted for multiple comparisons using the Bonferroni correction.Data are presented as the P-values for each parameter.The p-values were adjusted for multiple comparisons using the Bonferroni correction.T1 :post induction; T2:immediate post pneumoperitoneum; T3:0.5 hours post pneumoperitoneum; T4 :1 hour post pneumoperitoneum; T5:1.5 hours post pneumoperitoneum; T6:end of pneumoperitoneum; PEEP:positive end-expiratory pressure; Ppeak:peak inspiratory pressure; Plat:plateau pressure; Pmean:mean pressure; Crs:respiratory compliance; Raw:airway resistance; ΔP:driving pressure.

**Supplementary Table 3: Intra-group situation of the 10cmH_2_O PEEP group at different time poins .**

| Time Point | *P*-value | | | | | |
| --- | --- | --- | --- | --- | --- | --- |
|  | **Crs** | **Raw** | **Ppeak** | **Pmean** | **Plat** | **ΔP** |
| T1-T2 | 0.016 | 0.028 | 0.018 | 0.101 | 0.009 | 0.006 |
| T1-T3 | 0.000 | 0.000 | 0.000 | 0.000 | 0.000 | 0.000 |
| T1-T4 | 0.000 | 0.000 | 0.000 | 0.000 | 0.000 | 0.000 |
| T1-T5 | 0.000 | 0.000 | 0.000 | 0.000 | 0.000 | 0.000 |
| T1-T6 | 1.000 | 1.000 | 0.603 | 0.101 | 0.886 | 0.465 |
| T2-T3 | 0.025 | 0.315 | 0.029 | 0.865 | 0.031 | 0.042 |
| T2-T4 | 0.001 | 0.637 | 0.001 | 0.006 | 0.001 | 0.002 |
| T2-T5 | 0.007 | 0.634 | 0.001 | 0.003 | 0.003 | 0.003 |
| T2-T6 | 0.715 | 0.050 | 1.000 | 1.000 | 1.000 | 1.000 |
| T3-T4 | 1.000 | 1.000 | 1.000 | 1.000 | 1.000 | 1.000 |
| T3-T5 | 1.000 | 1.000 | 1.000 | 0.963 | 1.000 | 1.000 |
| T3-T6 | 0.000 | 0.000 | 0.000 | 0.421 | 0.000 | 0.000 |
| T4-T5 | 1.000 | 1.000 | 1.000 | 1.000 | 1.000 | 1.000 |
| T4-T6 | 0.000 | 0.000 | 0.000 | 0.002 | 0.000 | 0.000 |
| T5-T6 | 0.000 | 0.000 | 0.000 | 0.001 | 0.000 | 0.000 |

Data are presented as the P-values for each parameter.The p-values were adjusted for multiple comparisons using the Bonferroni correction.Data are presented as the P-values for each parameter.The p-values were adjusted for multiple comparisons using the Bonferroni correction.T1 :post induction; T2:immediate post pneumoperitoneum; T3:0.5 hours post pneumoperitoneum; T4 :1 hour post pneumoperitoneum; T5:1.5 hours post pneumoperitoneum; T6:end of pneumoperitoneum; PEEP:positive end-expiratory pressure; Ppeak:peak inspiratory pressure; Plat:plateau pressure; Pmean:mean pressure; Crs:respiratory compliance; Raw:airway resistance; ΔP:driving pressure.
